# Evaluation of selected polychlorinated biphenyls (PCBs) congeners and dichlorodiphenyltrichloroethane (DDT) in fresh root and leafy vegetables using GC-MS

**DOI:** 10.1038/s41598-018-36996-8

**Published:** 2019-01-24

**Authors:** Olatunde S. Olatunji

**Affiliations:** School of Chemistry and Physics, University of KwaZulu-Natal, Westville, Durban South Africa

## Abstract

Persistent organic pollutants (POPs) are dangerous and toxic pollutants that may cause adverse effects on human and animal health, including death. POPs such as polychlorinated biphenyls (PCBs) and pesticides are subtly released into the environment from industrial and agricultural use. Global circulation is due to their trans-boundary transport capacity, contingent on aerodynamic and hydrological properties. Plants have capacity to take-up POPs, and these bio-magnify along heterotrophic transfer pathways. In this study, levels of selected 6-PCB congeners and 3- DDTs in some leaf and root vegetables were investigated. Leaf and root vegetables were collected from different horticultural farms areas in Cape Town. The 6-PCBs and 3-DDTs were recovered from the samples using solid phase extraction(SPE), followed by GC-MS analysis. The ΣPCBs and ΣDDT (on-whole basis), were ranged: 90.9–234 *n*g/g and 38.9–66.1 *n*g/g respectively. The 3-PCBs and 6-DDTs levels were slightly higher in leaf vegetables compared to root vegetables. The detection of PCBs and DDTs in the vegetables suggest the probable use of PCBs containing pesticides. Although the observed concentrations were below the WHO maximum residue limits, consumption of such contaminated leaf and root vegetables portend a health risk.

## Introduction

Persistent organic pollutants (POPs) such as pesticides and polychlorinated biphenyls (PCBs), are synthetic substances produced for a wide range of sanitary health, agricultural and industrial uses^1^. They may also be unintentionally, generated as by-products of activities involving combustion^1^.

Nearly all POPs are characterized by poor water solubility, hence their resistance to decomposition. This results in their environmental persistence over a long time. They may therefore accumulate in soil, partitioned between water and sediment, or exponentially bio-accumulate up in the food chain^2,3^. Ritter *et al*.^4^ reported that, compounds such as pesticides and PCBs can persist and bio-accumulate by factors up to 70,000 fold in the environment. Low vapour pressure POPs can volatilize into air at ambient temperatures, and undergo transboundary atmospheric transfer reaching far destinations, or re-condensed at cooler temperatures.

Exposure to low concentrations of POP compounds can lead to severe toxic consequences and adverse health effects, such as cardiovascular effects (damage to immune and respiratory systems), damage to critical organs, teratogenic effects (birth defects), miscarriages and even death in humans and wildlife^5–7^. For instance, some pesticides have been reported to induce endocrine disruption with consequences on reproductive system, sex-linked disorders, and shortened lactation in nursing mothers’^8,9^. Carcinogenic and mutagenic responses^10,11^, as well as certain neurophysiological effects, such as attention deficits, learning disorders, behavioral problems including increased aggressivity, and poor gross and fine motor coordination have been linked to foetal exposure to POPs^7,12^.

Although different types of POPs exist, the polychlorinated biphenyls (PCB) and the organochlorine pesticide (OCP) compounds are among the most persistent, capable of magnification and with high mammalian toxicity. Polychlorinated biphenyls (PCBs) consist of 209 low vapour pressure, heat resistant, non-flammable congeners with high dielectric constants, used as heat exchange fluids or as insulating medium in electrical transformers and capacitors. Of these number, 13 congener compounds exhibit dioxin-like properties, and are reported to show extremely high toxicity^5,13,14^. While many of these chemicals pose serious environmental and human health risks on their own, the cumulative health effect that a combination of these chemicals may have is potentially of greater concern1^5–16^.

Persistent and bio-accumulative pesticides include many first generation organochlorine (OC) compounds such as 1, 1′-(2, 2, 2-Trichloroethane-1, 1-diyl)bis(4-chlorobenzene) (DDTs), dieldrin, toxaphene and chlordane. These compounds were largely used in agriculture for pests and weeds control, in order to maximize produce yield and thereby increase economic gains on the long run. Consequently, farm produce such as vegetable, perennial crops and many other pesticide-sprayed plants, take–up pesticides, storing them in plants cell lipid membrane, passing them from one heterotrophic level to another. According to Khan *et al*.^17^ soil-to-plant transfer is one of the major pathways of pesticides transport to shoots i.e. leaves and stems of plants grown on contaminated soil, or on soils irrigated with contaminated wastewater. There is therefore need to be concerned about the exposure and levels of PCBs and pesticides in vegetables, since they are a major diet and a transfer pathway.

Aside from the compromise of habitats and environment health, is the concern over the potential impact PCBs and DDTs can infringe on the world economy through food contamination. As these substances move up the food chain, their concentrations increase (bio-magnify), therefore animals atop food chain (fish, predatory birds, mammals and humans) may be exposed to several folds magnified concentrations of these chemicals. Findings from a study” on “Incremental Cancer Risk Assessment” conducted by Khillare *et al*.^18^ revealed that dietary intake of vegetables grown in the vicinities of thermal power plants is associated to carcinogenic health risks. Since vegetables can be potentially contaminated, dietary exposure could therefore elicit toxic responses, causing adverse effects including human and animal ill health and even death.

In 2012, the United Nation Environment Programme (UNEP) Scientific Committee of Experts (SC), targeted 22 POPs including the dirty dozen (Aldrin, dieldrin, DDT, endrin, heptachlor, chlordane, hexachlorobenzene (HCB), mirex, toxaphene, PCDD/Fs i.e. polychlorinated dioxins (PCDDs), polychlorinated dibenzofurans (PCDFs) and polychlorinated biphenyls (PCBs)) for elimination or restricted use, in order to minimize the contamination of food chain^19^. A strategy for human and animal protection is continuous environmental and food monitoring. While many analyses on water and fatty foods such as fish, milk, cheese and some groceries have been reported previously^20–24^, information concerning pesticide residues in short shelve life fresh vegetables and other food crops is scanty. Bouwman^25^ reported that despite the risks, they pose to human health, which are of great public concern information about the levels, distribution and fate of many POPs in soils, vegetables and other food samples is limited. Also, the assessment of POP residues especially the PCBs and OCPs in vegetables and other biological matrices in many Africa countries are limited, hence the scarcity of data on their levels, characterization and safety/health impact

In this study the levels of 6-PCBs congeners: (PCB_110–2, 2′, 4, 4′,6-pentachlorobiphenyl; PCB_118–2, 3′, 4, 4′, 5-pentachlorobiphenyl; PCB_138–2, 2′, 3, 4, 4′, 5-hexachlorobiphenyl; PCB_149–2, 2′, 3, 4′, 5′, 6-hexechlorobiphenyl; PCB_153–2, 2′,4, 4′,5, 5′-hexachlorobiphenyl; PCB_180 (2, 2′,3, 4, 4′, 5, 5′-heptachlorobiphenyl) and 3-DDTs (4, 4′-dichlorodiphenyltrichloroethane (DDT), 4, 4′-dichlorodiphenyldichloroethylene (DDE) and 4, 4′-dichlorodiphenyldichloroethane (DDD)) residues were investigated in fresh leaf (spinach, cabbage, lettuce, dhanial, celery, parsley and kale) and root vegetables (carrots, beetroot, radish, leeks, spring onion, cauli flower, turnip and broccoli) using GC-MS. This is in order to evaluate and characterize plant food (especially vegetables) health status vis-a-viz their occurrence levels, as well as set a benchmark in policy for periodic monitoring and guideline thresholds for human, animals and environmental health and safety.

## Methodology

### Chemicals and standard reference materials

Acetonitrile, acetone and acetic acid of analytical grade, purity > 98% from Sigma Aldrich used for the analysis. High purity standards (≫99.9%) used for instrumental calibration were purchased from Restek Inc. Internal standards, deuterated pp’DDT-d8 was purchased from Sigma Aldrich. Pre-packed solid phase cleanup kits (50 mL Teflon centrifuge/extraction tube and 15 mL Teflon centrifuge/clean-up frits tubes containing polymeric reverse phase (PRP) column) were obtained from phenomenex.

### Instrument calibration

Stock solutions of PCBs_110, _118, _138, _149, _153, _180, DDT, DDE and DDD standards were prepared to achieve 10 *µ*g/mL by diluting 1 mL, 1000 *µ*g/mL in 100 mL volumetric flask. Thereafter, cocktail of working calibration standards of 25, 50, 100, 500 and 1000 *n*g/mL were prepared by successive (serial) dilution in 100 mL volumetric flask to obtain a linear regression of five-point multi-component calibration standards of 8-mix. At initial, the standards of each of the analytes of the 6-PCB congeners and 3-DDTs were individually injected in six replicate measurements each to determine their retention time and characteristic fragment patterns. Thereafter, cocktail of each concentration levels of the working standards was injected on-line the GC-MS in selected reaction monitoring (SRM) mode. The integrated peak areas (signal) in response to gradient of standards of each of the measured analytes (chromatogram) was used in the identification of the analytes, while quantitation was by external calibration.

### Surrogate (Internal Standard) Recovery

Also, about 200 µL of surrogate internal standard solution of 4, 4′-DDT_d8 at low, medium and high concentrations was added to about 200 mL, 5% methanol in deionized water. The surrogate spiked solutions were allowed to equilibrate and treated using the same sample treatment procedures. Each concentration level was prepared and injected in triplicate as recommended by Camino-Sanchez *et al*.^26^. According to Sloan *et al*.^27^ an internal standard recovery of 60–120% should be achieved for analytes intended for GC-MS analyses.

#### Analytical homogeneity of sample replicates and certified reference materials (CRM)

Analytical homogeneity (<15% RSD) of the sample results was tested by investigating the reproducibility of replicate measurements of samples at specified intervals/frequency, (10 samples), and that of replicate measurements of certified reference materials (CRM-IAEA-140-OC) during analysis.

#### Sample collection

Farm soils, leaf vegetables (spinach – *Spinacia oleracea*, cabbage – *Brassica oleracea*, lettuce – *Lactuca sativa*, dhanial – *Coriandrum sativum*, celery – *Apium graveolens*, parsley – *Petroselinum crispum*, and kale – *Brassica oleracea*), and root vegetables (carrot – *Daucus carota*, cauliflower – *Bassica oleracea*, radish – *Raphanus raphanistrum*, broccoli – *Brassica oleracea*, turnip – *brassica rapa*, leek – *allium ampeloprasum*, and spring onion – *allium cepa*), were collected from seven formal and informal farms geo-referenced 18.5304588 E and –34.0154588 SS, around Cape Town, between January and October 2017 on a monthly basis.

The leafy vegetables were stripped into furnace (Gallenkamp) fired (550 °C for 1 hr) dilute nitric acid pre-cleansed/swabbed aluminum foil; root vegetables were collected by slicing into furnace fired dilute nitric acid pre-cleansed aluminum foils; and the soils were pooled from farm top soil (0–15 mm) into nitric acid pre-cleansed aluminum foil using a stainless steel hand trowel. Each of the samples collected, were then placed in well-labelled zip plastic bags in order to isolate the sample from each other to prevent cross contamination, and thereafter place in a cooler with Ice Park for onwards transfer to the laboratory. The collected samples were frozen at −20 °C in the refrigerator until processing. All samples were process within 48 hr. of collection

### Sample preparation and extraction of pesticides from vegetable samples

The leaf and root vegetables were split into two; with a split washed in clean water before air-drying, while the other split was dried directly without washing and dried at ambient laboratory conditions. The farm soil samples were also air dried in the laboratory at ambient conditions. After drying, each of the leaf and root vegetables were crushed into fines, while the soil samples were pulverized to <2 mm particle size.

Extraction of the analytes was carried out using the pre-packed solid phase extraction column. About 5 g of homogenized vegetable sample was weighed into centrifuge tube. Thereafter, 100 *μL* of internal standard (10 ppm) was added to the homogenate, followed by 10 ml of Milli-Q-water and 10 ml of acetonitrile acidified with 10% of acetic acid. The mixture was thoroughly homogenized on a vortex at a revolution of 2000 rpm for 2–3 min. The mixture was allowed to stand for 15 minutes. Thereafter, approximately 6 g MgSO_4_ and 1.5 g NaCl was then added to the homogenate, and thoroughly mixed and vortexed for another 3 minutes. The homogenate mixture was allowed to stand for 5 min, and then centrifuged at a revolution of 2 000 rpm for about 5 minutes. The PCBs and DDT mass extract in acetonitrile supernant was decanted from the solid residue and cleaned up in the PRP pre-packed cleanup column. The recovered extract in acetonitrile was concentrated <0.5 mL using the CentriVap concentrator under nitrogen stream and reconstituted to 1 mL in acetonitrile for analysis.

### Extraction of pesticides from farm soil samples

About 5 g each of the pulverized soil sample was weighed into centrifuge tube. This was followed by the addition of mixture of 10 mL of Milli-Q-water and 10 mL of acetonitrile acidified with 10% of acetic acid. The resulting mix was homogenized on a vortexed at 2000 rpm for 2 min. Approximately 5 g MgSO_4_ and 1 g NaCl were added to the homogenate afterwards, and vortexed again for another 2 min. About 20 mL of mixture of high purity acetonitrile and acetone (60:40) was then added to homogenate, and left to equilibrate on a horizontal shaker for 10 min, and afterwards allowed to stand for 20 min. The PCBs and DDT mass extracts in acetonitrile and acetone mix was decanted from the soil residue, and concentrated to near dryness using a CentriVap concentrator under nitrogen stream, and reconstituted to 1 mL in acetonitrile for analysis

### Analysis PCB and DDT Analyses and data analysis

Congener specific analysis was conducted on the recovered analytes in acetonitrile-acetone extracts, respectively pooled from the fresh leaf and root vegetables, and top soil samples to identify and quantify residues of PCBs: 110 (2, 2′, 4, 4′, 6-pentachlorobiphenyl), 118 (2, 3′, 4, 4′, 5-pentachlorobiphenyl), 138 (2, 2′, 3, 4, 4′, 5-hexachlorobiphenyl), 149 (2, 2′, 3, 4′, 5′, 6-hexechlorobiphenyl), 153 (2, 2′, 4, 4′, 5, 5′-hexachlorobiphenyl), and 180 (2, 2′, 3, 4, 4′, 5, 5′-heptachlorobiphenyl), and DDTs: dichlorodiphenyltrichloroethane (4, 4′-DDT), dichlorodiphenyldichloroethylene (4, 4′-DDE), and dichlorodiphenyldichloroethane (4, 4′-DDD).

Analysis were performed using a gas chromatograph (Agilent Technologies; 6890N) fitted with an auto sampler and coupled a mass spectrometry detector (5975) (GC-MSD). About 1.0 μL each of the samples were injected in the splitless mode, in-stream of helium carrier gas over a DB-5 (5%-phenyl-95%-dimethylpolysiloxane) capillary column (Waters; 30 m × 0.25 mm i.d., 0.25 *μ*m film thickness), at a flow rate of 3 mL/min, with nitrogen as make-up gas. The injector port temperature was kept at 250 °C, while the sample streams over the column in gradient of temperature set initially at 70 °C and maintained for 2 min holding time. The temperature was afterwards ramped at a rate of 25 °C/min to 180 °C, held for 3 min; 15 °C/min to 250 °C, held for 2 min, and then 8 °C/min to 290 °C, held for 5 min.

Fractional eluents from the column reached the mass selective spectrometer detector (MSD) quadrupole via the transfer line set at a temperature of 300 °C. Ionization source temperature was set at 300 °C, and operated in the negative electrospray ionization (ESI), with argon collision scanning, operated in the selected reaction monitoring (SRM) mode at capillary voltage set at 3 kV, while the ions data were acquired via selected ion monitoring (SIM) with two characteristic ions. Quantification of the analytes was performed using the external standard technique.

### Statistical Analyses

The data obtained were subject to evaluation using descriptive statistics and one-way analysis of variance (ANOVA) with consequent Duncan test (Statistica 7.0, StatSoft). The results were interpreted based on homogeneous groups at p < 0.05 significance level

## Results and Discussions

### Optimization and validation of method

#### Method validation parameters

The efficiency of the GC-MS method for the determination of the 6-PCBs and 3-DDTs was validated based on the guidance criteria of the International Conference on Harmonization^28^. Validation parameters such as limit of detection (LOD) and limit of quantification (LOQ) of the method were evaluated using blank and fortified replicates (n = 10) for all analytes in each matrix mix.

The retention time and their SRM m/z characteristic fragmentation pattern of each of the individual analyte was determined by means of six online injections. The MS/MS chromatograms for the elution of each of the injected individual analytes: 4.4′-DDE, 4.4′-DDD, 4.4′-DDT, PCB_110, PCB_118, PCB_138, PCB_149, PCB_153 and PCB_180 indicated their retention time (supplementary file).

The retention time for the elution of each of the 6-PCBs and 3-DDTs analytes, and their characteristic SRM m/z fragmentation pattern for the qualifying and quantifying ions, parent and product masses as well as their collision energies were recorded

Linearity and linear range were assessed using the calibration curve obtained from the plot of peak count signal for the injection of fortified gradient standard’s prepared by spiking acetone with known concentrations of the analytes at working concentrations range from 25 to 1000 *n*g/mL. The coefficient of regression (R^2^) obtained for the concentration intervals between 25 *n*g/mL and 1000 *n*g/mL were all greater the 0.00 (R^2^ > 0.99) (Table 1).

**Table 1:** Linearity, linear range and coefficient of regression characteristics of 6-PCBs and 3-DDTs of optimized instrument (calibration).

	Retention time (RT)	Regression equation	Coefficient of regression (R^2^)
4, 4′-DDE	18.61	Y = 83900*x − 2.326 × 10^−6^	0.9990
4, 4′-DDD	19.29	Y = 111684*x − 4.424 × 10^−6^	0.9969
4, 4′-DDT	19.46	Y = 670104*x − 2.653 × 10^−6^	0.9943
2, 3, 3′, 4′, 6-PCB (PCB_110)	18.75	Y = 43969*x + 1542 × 10^−6^	0.9951
2, 3′, 4, 4′, 5-PCB(PCB_118)	19.30	Y = 75724.9*x + 2215 × 10^−6^	0.9995
2, 2′, 3, 4, 4′, 5′-HCB(PCB_138)	19.15	Y = 479124*x + 3043 × 10^−6^	0.9799
2, 2′, 3, 4′, 5′, 6-HCB(PCB_149)	19.65	Y = 604210*x + 6981 × 10^−6^	0.9892
2, 2′, 4, 4′, 5, 5′-HCB(PCB_153)	20.09	Y = 38926.8*x + 7963 × 10^−6^	0.9917
2, 2′, 3, 4, 4′, 5, 5′-HeCB (PCB_180)	21.31	Y = 24427.4*x + 8568 × 10^−6^	0.9968

Linear responses for the 3-DDTs gave coefficient of regression 0.9969, 0.9990 and 0.9943 for 4, 4′-DDD, 4, 4′-DDE and PCB_180 respectively, while the coefficient of regression for the 6-PCBs were, 0.9951, 0.9995, 0.9799, 0.9892, 0.9917, and 0.9968 for PCB_110, PCB_118, PCB_138, PCB_149, PCB_153 and PCB_180 respectively

The LODs for each analyte was thereafter obtained as the quotient of three multiples of the standard deviation (*σ*) of experimental blank (noise) with respect to the slope (m) of calibration curve of gradient concentration plot $({\rm{LOD}}=\frac{3{\boldsymbol{\sigma }}}{{\boldsymbol{m}}}),$ while the LOQ was determined as ten multiples of the standard deviation of the experimental blank (noise), i.e. LOQ = 10 x std. dev.). The LOD (*n*g/mL) of PCB_110, PCB_138, PCB_118, PCB_149, PCB_153, and PCB_180 were: 0.075, 0.018, 0.034, 0.022, 0.022 and 0.027 respectively, while the LOD of 4, 4′-DDD, 4, 4′-DDE, and 4, 4′-DDT were 0.052, 0.028, and 0.016 *n*g/mL respectively (supplementary file).

### Precision and Accuracy

Comparable recoveries were achieved for all the analytes (supplementary file). The average recovery of the labelled internal standard 4, 4′-DDT_d8 ranged between 72% and 90%, while those for the 3-DDTs and 6-PCBs ranged between 68 and 94%. Intra-class correlation (ICC) used for the comparison of influences of intra-individual and inter-individual variances in the recoveries of analytes in spiked and unspiked samples, on the whole variance observed for the DDTs and PCBs measurements were in agreement with the of The Two-Way Random Absolute ICC values.

### Concentrations levels of DDTs and PCBs in leafy and root vegetable and soil samples

The concentration profiles of the 6-PCBs congeners and the 3-DDTs in the leafy vegetables, root vegetables and farm soils varied. This could be attributed to several factors including selective soil abstraction and translocation, variation in aerials uptake based of amount reaching plant aerial parts, stability of PCB and DDTs, pesticide dissipation and degradation (half-life) in soil, biological factors such as microbial population, types, and characteristics, weather and soil condition. The concentrations (on-whole basis) of the residues of the 3-DDTs and 6-PCBs in the leafy vegetables was found to be generally variable (Table 2).

**Table 2:** Concentrations (*n*g/g) of 3-DDTs and 6-PCBs in leaf and root vegetables samples.

Leaf veg	4, 4-DDD	4, 4-DDE	4, 4-DDT	Σ_3_DDTs	PCB_110	PCB_118	PCB_138	PCB_149	PCB_153	PCB_180	Σ_3_PCBs
Spinach	10.1 ± 5.58	10.5 ± 4.74	18.3 ± 6.63	38.9	27.7 ± 15.7	20.5 ± 13.10	13.1 ± 6.75	12.0 ± 4.83	15.2 ± 5.83	13.6 ± 4.73	102
Lettuce	20.7 ± 7.05	13.2 ± 4.90	28.2 ± 9.81	62.2	32.9 ± 16.5	26.8 ± 11.95	19.5 ± 8.06	20.4 ± 7.63	19.8 ± 7.34	18.4 ± 5.06	234
Celery	20.9 ± 8.84	13.5 ± 6.42	28.6 ± 8.56	63.1	21.3 ± 14.1	24.9 ± 12.15	17.5 ± 7.48	22.0 ± 6.90	21.1 ± 6.76	19.1 ± 7.85	126
Parsley	22.0 ± 7.80	14.9 ± 5.89	20.3 ± 6.27	57.2	31.4 ± 18.1	27.6 ± 13.97	19.9 ± 8.42	25.7 ± 7.85	24.6 ± 8.05	23.4 ± 8.02	153
Cabbage	12.5 ± 6.32	11.6 ± 5.08	21.1 ± 7.91	45.3	15.9 ± 10.5	18.8 ± 10.43	12.1 ± 7.70	17.0 ± 7.29	16.6 ± 5.75	15.3 ± 6.41	95.8
Dhanial	19.6 ± 6.74	13.5 ± 6.33	27.0 ± 8.05	60.5	34.5 ± 17.3	27.9 ± 12.67	17.9 ± 6.95	23.4 ± 6.41	22.4 ± 8.21	21.5 ± 7.62	148
Kale	20.2 ± 8.61	14.0 ± 7.02	27.9 ± 9.15	62.1	36.2 ± 18.5	24.8 ± 11.81	18.4 ± 9.90	23.9 ± 7.28	22.9 ± 6.34	20.9 ± 8.04	147
Sum	126	91.2	171	389	200	171	117	145	142	132	1005
Mean	18.0	13.0	24.5	55.6	28.6	24.5	16.9	20.7	20.3	18.9	144
Std. dev.	4.69	1.48	4.40	9.60	7.45	3.55	3.10	4.71	3.42	3.46	45.8
**Root veg**	4, 4-DDD	4, 4-DDE	4, 4-DDT	Σ_3_DDTs	PCB_110	PCB_118	PCB_138	PCB_149	PCB_153	PCB_180	Σ_3_PCBs
Broccoli	14.6 ± 3.07	11.6 ± 2.19	20.1 ± 8.19	46.4	29.8 ± 10.4	21.2 ± 9.58	14.4 ± 7.90	17.7 ± 8.29	17.0 ± 6.82	15.6 ± 8.30	116
Redish	12.7 ± 4.18	11.9 ± 2.84	21.8 ± 5.32	46.3	23.2 ± 11.1	20.4 ± 8.82	15.2 ± 7.13	17.8 ± 7.90	17.7 ± 5.49	15.7 ± 7.52	110
Couliflower	11.2 ± 2.55	10.4 ± 4.17	19.6 ± 6.86	41.2	13.9 ± 7.26	18.9 ± 7.25	12.9 ± 8.42	16.1 ± 8.84	15.6 ± 5.87	13.4 ± 7.35	90.9
Leek	16.5 ± 4.71	12.2 ± 3.26	24.2 ± 9.03	52.8	30.8 ± 12.2	23.5 ± 9.74	14.9 ± 9.11	17.9 ± 6.38	17.4 ± 7.25	15.6 ± 8.63	144
Turnip	15.6 ± 3.18	12.1 ± 2.72	22.6 ± 7.38	50.3	13.6 ± 9.61	21.0 ± 8.02	15.3 ± 6.86	17.4 ± 8.05	17.1 ± 8.02	15.7 ± 7.20	100
Spring onion	18.9 ± 5.21	13.4 ± 3.09	26.2 ± 8.75	58.6	32.3 ± 17.1	25.5 ± 8.79	16.9 ± 7.10	19.7 ± 7.82	19.5 ± 9.71	18.3 ± 7.58	132
Carrot	20.1 ± 5.09	14.7 ± 2.95	31.4 ± 9.94	66.1	35.6 ± 12.8	29.1 ± 9.07	18.9 ± 8.01	22.7 ± 9.74	22.2 ± 9.95	21.2 ± 9.31	150
Sum	110	86.2	166	362	179	160	109	129	127	116	843
Mean	15.7	12.3	23.7	51.7	26.6	22.9	15.5	18.5	18.1	16.5	120
Std. dev.	3.12	1.36	4.09	8.42	8.89	3.49	1.91	2.15	2.13	2.49	22.3

**Figure 1:**
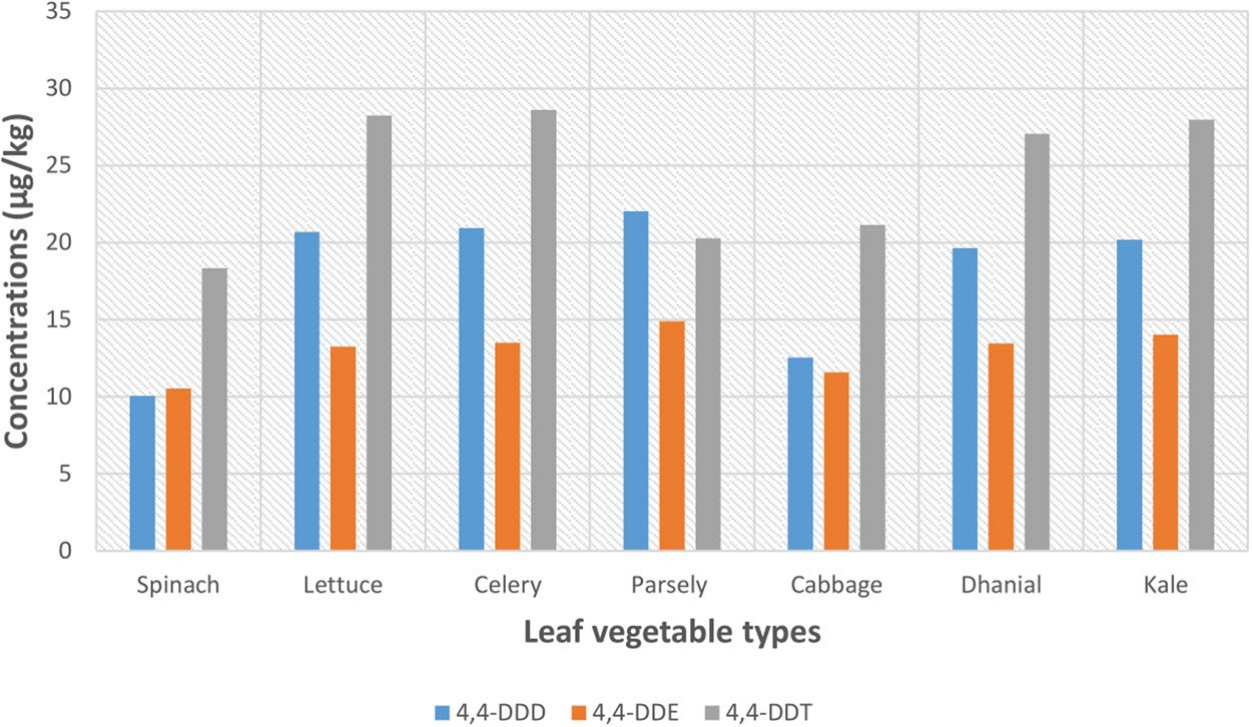
Composition profile of DDT and its metabolites DDD & DDE in leaf vegetables.

The highest DDTs (i.e. 4, 4 – DDD, and 4, 4 – DDE) concentrations was observed in parsley, with mean concentration of 22.0 ± 7.80 *n*g/g and 14.9 ± 5.89 *n*g/g, respectively, while celery vegetable had the highest concentration of 4, 4 – DDT (28.6 ± 9.81 *n*g/g) (Fig. 1).

The lowest concentration of Σ_3_DDTs was observed in spinach (Σ_3_DDTs, 38.9 *n*g/g), while the highest was noted in celery (Σ_3_DDTs, 63.1 *n*g/g). The observed low concentrations may be a consequence of the ban on the use of DDT OCPs. However, DDT somehow finds its way into the South African market for crops and animals protection purposes, as well for residual indoor spraying to control vector^29^. Aside from this, the occurrence of DDT in vegetables may also be due to aerial deposition of leaks and other usage source dissipated airborne suspended DDTs, on soil and vegetation, especially in near vicinities where soil is intensively used for farming, and their persistence (owing to their hydrophobic properties) as a result of usage deposition from the past.

**Figure 2:**
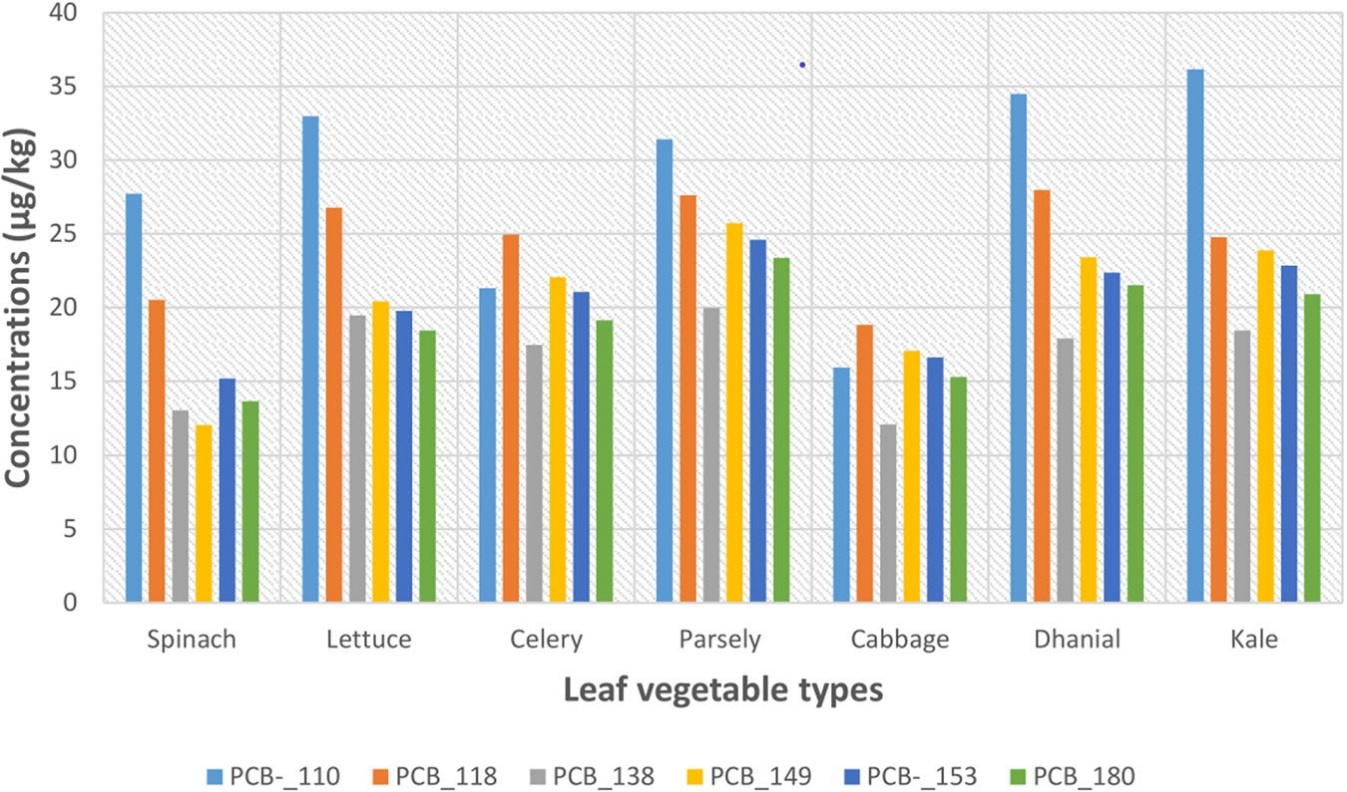
Composition profile of 6-PCBs congeners in the leaf vegetable.

The concentrations of the PCB congeners were generally variable, with detected mean concentrations ranged: PCB_110, 15.9 ± 10.5–36.2 ± 18.5 *n*g/g; PCB_118, 18.8 ± 10.4–27.9 ± 12.7 *n*g/g; PCB_138, 12.1 ± 7.70–19.9 ± 8.42 *n*g/g; PCB_149, 17.0 ± 7.29–23.9 ± 7.28 *n*g/g; PCB_153, 16.6 ± 5.75–24.6 *n*g/g and PCB_180, 13.6 ± 4.73–23.4 ± 8.02 *n*g/g in all the tested leafy vegetables (Fig. 2).

The concentrations of PCB_110 congener in all leafy vegetables (ΣPCB_110, 200 *n*g/g) was found to be the highest while the concentrations of PCB_138 congener (ΣPCB_138, 119 *n*g/g) was found to be the lowest.

However, the observed concentrations of the ΣPCBs and ΣDDTs in the leafy vegetables were below the maximum residue limits as recommended by world health organisation^4,5^. This suggest that the probability of human and animal health compromise associated to the consumption of vegetables from the informal and formal farms is low. While human and animal exposure via vegetable consumption poses very little health risk, fauna populations in immediate habitats within residues occurrence areas may be at risk.

**Figure 3:**
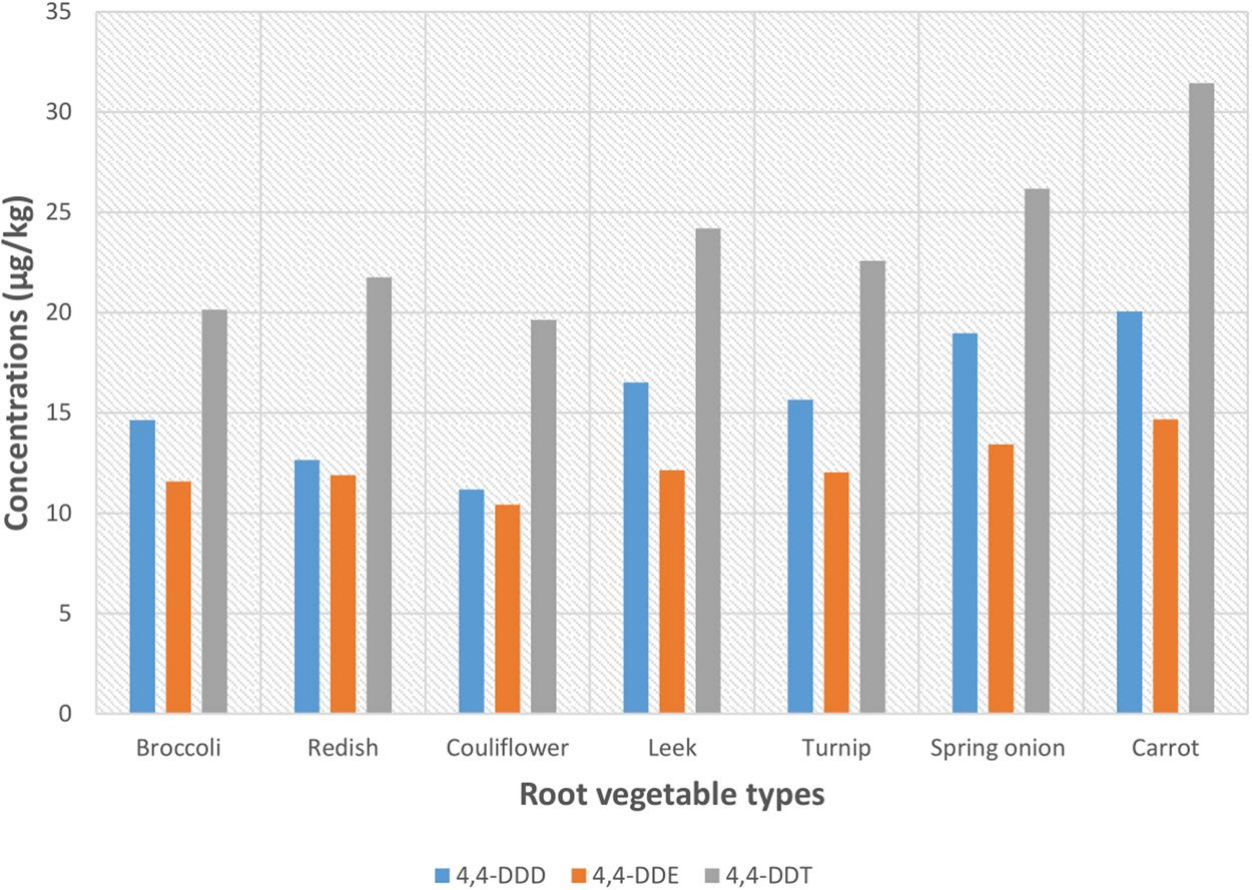
Mean concentrations of 4.4-DDTs in the tested vegetables collected in different farms lands.

The mean concentrations (*ng/*g) of 4, 4-DDT in the root vegetables i.e. carrots, spring onion, leek, turnip, reddish, broccoli and cauliflower were: 31.4 ± 9.94, 26.2 ± 8.75, 24.2 ± 9.03, 22.6 ± 7.38, 21.8 ± 5.32, 20.1 ± 8.19 and 19.6 ± 6.86, respectively. The levels (*n*g/g) of metabolites 4.4-DDD were: carrots, 20.1 ± 5.09; spring onion, 18.9 ± 5.21; leek, 16.5 ± 4.71; turnip, 15.6 ± 3.18; broccoli, 14.6 ± 3.07; reddish, 12.7 ± 4.18 and couli flower, 11.2 ± 2.55; and 4.4-DDE were: carrots, 14.7 ± 2.95; spring onion, 13.4 ± 3.09; leek, 12.2 ± 3.26; turnip, 12.1 ± 2.72; reddish, 11.9 ± 2.84; broccoli, 11.6 ± 2.19 and cauliflower, 10.4 ± 4.17. (Fig. 3).

Carrots probably have a slightly higher capacity to accumulate all 3-DDTs, since and contained more 4, 4-DDE, 4, 4-DDD and 4, 4-DDT, compared to others root vegetables; while couliflower had the least average concentration of 4, 4-DDE, 4, 4-DDD and 4, 4-DDT. The mean concentrations of the 3-DDTs ranged: DDT, 19.6 ± 6.86–31.4 ± 9.94 *n*g/g; DDE, 10.4 ± 4.17–14.7 ± 2.95 *n*g/g; and DDD, 11.2 ± 2.25–20.1 ± 5.09 *n*g/g, respectively, in cauliflower and carrot respectively.

**Figure 4:**
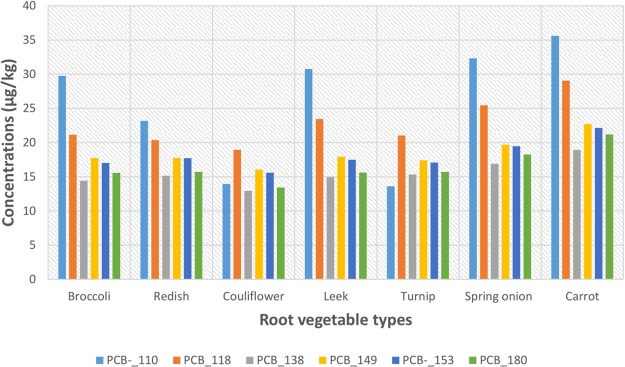
Mean concentrations of 6-PCBs congeners in the root vegetables collected from different farms lands.

In addition, cauliflower had the least concentrations of all PCB congeners (except for PCB_110 levels in turnip) in all the root vegetables tested, while carrots contained the highest (Table 2). The mean concentrations (µg/kg) were: congener PCB_110: carrots, 35.6 ± 12.8; spring onion, 32.3 ± 17.1; leek, 30.8 ± 12.2; broccoli, 29.8 ± 10.4; reddish, 23.2 ± 11.1; turnip, 13.6 ± 9.61 and cauliflower, 13.9 ± 7.26 (Fig. 4).

The mean concentration of the 6-PCB congener were ranged between: 13.6 ± 9.61 and 35.6 ± 12.8 *n*g/g for PCB_110; 18.9 ± 7.25 and 29.1 ± 9.07 *n*g/g for PCB_118; 12.9 ± 8.42 and 18.9 ± 8.01 *n*g/g for PCB_138; 16.1 ± 8.84 and 22.7 ± 9.74 *n*g/g for PCB_149; 15.6 ± 5.87 and 22.2 ± 9.95 *n*g/g for PCB_153 and 13.4 ± 7.35 and 21.2 ± 9.31 *n*g/g PCB_180, respectively, in cauliflower and carrots (Table 2). The sum of the concentration of the measured PCBs (Σ_6_PCBs) reached 843 *n*g/g, and was slightly elevated in comparison to the sum of the measured DDT and its DDD and DDE metabolites (Σ_3_DDT), which total, 362 *n*g/g concentration. The observed concentrations of the 3-DDTs and 6-PCBs in the different root vegetables though variable, are consistent with findings from other studies^30–32^. The observed mean concentrations of the POPs in leaf vegetables were generally slightly higher than in the root vegetables, except for DDE.

The concentrations of the 3-DDTs and 6-PCBs in farm soils also varied across all 7-farms (Table 3).

**Table 3:** Concentrations (*n*g/g) of 6-PCBs and 3-DDTs in farm soils (d.w.).

Top soil	4, 4-DDD	4, 4-DDE	4, 4-DDT	PCB_110	PCB_118	PCB_138	PCB_149	PCB_153	PCB_180
FML_1	35.1	41.9	58.5	46.1	56.8	70.9	85.2	95.4	120
FML_2	30.8	36.1	50.6	46.2	56.9	71.1	66.7	78.5	92.7
FML_3	26.8	32.2	44.7	41.8	51.4	64.2	41.9	47.4	55.8
FML_4	28.6	47.3	66.3	46.6	57.4	71.7	86.6	98.5	123
FML_5	37.5	45.2	62.6	45.3	55.7	69.7	40.9	46.3	54.5
FML_6	45.0	54.6	75.8	51.2	62.9	78.7	93.1	103	129
FML_7	48.2	55.8	80.3	59.0	72.6	90.8	102.5	114	142

d.w. - dry weight.

There were strong correlations (ϒ^2^ > 0.53 – ϒ^2^ > 0.78) between DDTs and PCBs levels detected in each of the root vegetable types and soil concentration levels. Multivariate regression model revealed also strong relationships between ΣDDTs and ΣPCBs concentration levels in the farm soils, farm site locations, and environmental pollution.

However, the observed concentration levels are somewhat higher than reported in Australia, Mexico and Poland, but lower than reported in Russia and Spain^30–32^. Since DDT and it metabolites are listed among the Stockholm Convention dirty 12^19^, regulation vis-a-viz legislation should be enforced to reduce their use and exposure sources of exposure in SA.

### Retention and fate of DDTs and PCB congeners

Comparison of leaf and root vegetables concentration of the 3-DDTs and 6-PCBs, revealed a slightly higher levels in all leaf vegetables than in root vegetables; with mean concentrations of PCB 110; PCB 118, PCB 138, PCB 149, PCB 153 and PCB 180; 28.6 *n*g/g; 24.5 *n*g/g; 16.9 *n*g/g; 20.7 *n*g/g; 20.3 *n*g/g and 18.9 *n*g/g, respectively, and 26.6 *n*g/g; 22.9 *n*g/g; 15.5 *n*g/g; 18.5 *n*g/g; 18.1 *n*g/g and 16.5 *n*g/g, respectively. Mean concentrations of DDD, DDE and DDT in leaf and root vegetable are 18.0, 13.0, 24.5 *n*g/g and 15.7, 12.3, 23.7 *n*g/g, respectively. The difference in levels detected in the leaf and root vegetables were not significantly different (P < 0.05). This suggests that the vegetables may probably have retention holding capacities that could facilitate ease of plant contamination vis-a-viz uptakes. Although, the differences in the levels observed in the leaf and root vegetables are not significant (p < 0.05), the detected concentrations were significantly lower than observed in the farm soils. It is however not clear, whether the total ΣDDTs and ΣPCBs are at levels that could initiate any stress on the plants, or even toxic responses. This depends on factors such as the magnitude of contaminants concentration, age of DDTs and PCBs in the soils, and plants uptake and accumulation capacity. The C-C, C-H as well as C-Cl intra-atomic bonds in many DDTs and PCBs confers on them such properties that facilitates their high molecular weight, low vapour pressure, low polarity, low water solubility, resistance to hydrolysis, and poor microbial activities. This may therefore result in their environmental stability/persistence with a potential to induce toxicity on different organisms^33–35^ and also governs the dynamics of their uptake pattern.

Since DDTs and PCB congeners show resistance to breakdown, the metabolism of these substances is very slow. Thus, the proportion of their metabolites will vary with the parent compounds in different media^36,37^. According to Polder *et al*.^38^ the ratio between 4, 4 – DDT and its metabolites 4, 4 – DDE and 4, 4 – DDD “is often used to describe recent or historic exposure of DDT product to the environment”. Low ratios of 4.4 – DDE/4.4 – DDT and 4.4 – DDD/4.4 – DDT in different matrices is indicative of recent use of DDT, high ratios of 4.4 – DDE/4.4 – DDT and 4.4 – DDD/4.4 – DDT indicate the occurrence of aging DDT after long time use, with increase in residues of the adduct metabolite products of environmental decomposition. The ratio of 4.4 – DDE/4.4 – DDT and 4.4 – DDD/4.4 – DDT in the leafy vegetables ranged between 0.59 in cabbage and dhanial, and 0.72 in spinach, and 0.43 in parsley and 0.61 in lettuce, respectively. In the root vegetables, the ratio of 4.4 – DDE/4.4 – DDT and 4.4 – DDD/4.4 – DDT were ranged 0.59 in turnip and spring onion, to 0.72 in broccoli and cauliflower, and 0.43 in leek to 0.61 in radish. The 4.4 – DDE/4.4 – DDT and 4.4 – DDD/4.4 – DDT ratio observed in the leaf and root vegetables suggest slight aging from possible recent use.

In addition, the capacity of the leaf and root vegetables to retain the 3-DDTs and 6-PCBs is indicative of their potential as a pathway for the translocation of these substances especially through the food chain. Results indicated variability in the ability of the vegetables to bio-accumulate the DDTs and the PCB congeners. The leafy vegetables bioaccumulation factors (*BAF* = *Cp*/*Cs*; *Cp - concentration in aerial part of plant, and Cs - concentration in soil*) for the 3-DDTs and 6-PCBs in leaf vegetables ranged from 0.25 for 4, 4-DDT in dhanial and kale to 0.78 for 4, 4-DDD in celery, and from 0.11 for PCB_180 in spinach to 0.71 for PCB_110 in lettuce. The bioaccumulation factor of the root vegetables on the other hand ranged from 0.25 for 4, 4-DDE in spring onion to 0.58 for 4, 4-DDD in leek and 0.13 for PCB_180 in leek and broccoli to 0.66 for PCB_110 in Leek.

Apparently, all the leafy vegetables showed higher capacity for the DDTs and PCB congeners than the root vegetables. The less chlorinated PCB congeners were readily taken-up by vegetables (higher BAF) compared with the more chlorinated PCBs (lower BAF). Similarly the bioaccumulation factor for metabolite 4, 4-DDD was higher in all vegetables compared with 4, 4-DDE and 4, 4,-DDT. This could be due to their solubility, though soil concentration levels were quite higher than observed in the leaf and root vegetables. Thus, heterotrophic transfers hold the likelihood of human and animal exposure, and this portends a potential for health compromise.

Generally, DDTs and PCB congeners occurred in all the farm soils, with intermittent detection of high concentration levels along discrete farm portions, while their concentration levels in the leaf and root vegetables were low. Our findings showed that vegetables and crop planted in farmlands within locations near residences and urban centers, especially informal farms in homes, may be exposed to leaked pesticides from home use and arrays other contaminants; many of which are classified as endocrine disruptors and neuro-degenerative substances. Therefore, there is a need for control by enacting and implementing regulatory policies that will curtail the use DDTs and PCBs containing products, as well as the release of DDTs and PCBs.

### Distribution of the 3-DDTs and 6-PCBs congeners

Evidence clearly indicate heterogeneity in the occurrence levels of the 3-DDTs and 6-PCBs detected in at the different farm sites. The concentration observed in the leafy vegetables and the root vegetables were also variable over farms. This might not be unconnected with the differential partition based on the soil characteristics and topographic inequalities of the farm plots. More also, the sources of the 3-DDTs and 6-PCBs into soil and their distribution pattern and pathways as well as uptake mechanism into the vegetables and perhaps other vegetation is not very clear. Channa^39^ reported that the occurrence of elevated levels of 4, 4′-DDE and 4, 4′-DDT may be associated or probably due to exposure to DDT contaminated foods, or food prepared using DDT contaminated wood. The higher concentration levels detected in soils may possibly be due to the large commercial and subsistence farming activities.

Thus, the presence of DDT and PCBs in soil could be a good indicator of deposition pattern due to the likely use of pesticides containing OCPs and some PCB congeners, for agricultural purpose such as pest control, increase crop yields, or contamination from long-range transport from diffuse sources afar. However, soil concentration of the sum of 3-DDTs and 6-PCBs were lower than reported in soil samples analyzed in Glasgow, Torino, Aveiro, Ljubljana and Uppsala with concentrations 22.0 µg/kg, 14.0 µg/kg, 7.90 µg/kg, 6.80 µg/kg, and 5.70 µg/kg respectively. Background values of 0.53 µg/kg was reported in upper agricultural field in Germany. The variabilities in the distribution profile of the 3-DDTs and 6-PCBs across the farm land soils, may be associated with the age of application/deposition, vapor pressure/volatilization potential and climatic influence.

The global concentrations of OCPs in all varieties of plants were estimated to range from 0.5 to 100 µg/kg dry weight^40^. The levels detected in the investigated vegetables in this study were within the global range, with the sum of the concentrations of the 3-DDTs being below the European Commission^41^ legally tolerated maximum residue level (MRL) of 500 µg/kg pesticide residues in food, leaf vegetables, herbs and edible flowers. The observed levels were consistent with the concentration of OCPs (mean concentration; 4 µg/kg) vegetables such as graviola, mullaca and balsamina in Bolivia and Peru^40^. An average OCPs concentration range of 0.18 ± 0.14 ng/g to 0.76 ± 0.43 ng/g was reported in India, while PCBs concentration ranged between <DL and 99.4 ng/g (13.4 ± 0.06 ng/g). Study results also agrees with Aichner *et al*. and Wang *et al*.^42,43^ for plants in the Kathmandu and Tibetan Plateau, China, respectively. Lower concentrations of PCBs were however reported in Vietnam, Romania, China, Mexico^44–47^, while higher levels noted in Turkey^48^.

## Conclusion

Concentrations of the 3-DDTs i.e. DDT and its metabolites DDD and DDE and 6-PCBs congeners in the 7-varieties of leafy vegetable and the 7-varieties of root vegetables collected from different farms were variable. The concentrations of these POPs were quantified with an allowed limit of detection (LOD) and the limit of quantification (LOQ) between 0.010–0.022 µg/L and 0.030–0.042 *n*g/mL respectively for the 3-DDTs and 6-PCB congeners. The sum of the concentration of the 3-DDTs in the leaf and root vegetables ranged between 41.2–66.1 *n*g/g, and 38.9–62.1 *n*g/g, respectively, while the 6-PCBs ranged between 90.9–149 *n*g/g, and 95.8–234 *n*g/g, respectively.

The observed concentrations of the 3-DDTs and 6-PCBs congeners, 110, 118, 138, 148, 153 and 180 were low. The concentrations of the tested POPs in all the leaf and root vegetables were all below the European Commission maximum residue levels and the maximum residue limits suggested by World Health Organization (WHO). Thus, the vegetables are relatively uncontaminated, and the occurrence of residues of the POPs may not be the result of POP pesticides use. The DDTs and PCBs in the vegetables and soil may be due to contamination arising from deposition of long ranged transported pesticide aerosol, incineration or split leaks from vector control residual indoor application from nearby residences and other sources.

## Supplementary information

Supplementary Tables
