# Supplementary material for: Evaluation of selected polychlorinated biphenyls (PCBs) congeners and dichlorodiphenyltrichloroethane (DDT) in fresh root and leafy vegetables using GC-MS
Source: Sci Rep. 2019 Jan 24;9:538. doi: 10.1038/s41598-018-36996-8 (PMC6345796; doi:10.1038/s41598-018-36996-8)
Supplement: Supplementary file 1 — Supplementary Tables [file 41598_2018_36996_MOESM1_ESM.pdf]

Evaluation of selected polychlorinated biphenyls (PCBs) congeners and dichlorodiphenyltrichloroethane (DDT) in fresh root and leafy vegetables using GC-MS

Olatunde S Olatunji

School of Chemistry and Physics
University of KwaZulu-Natal,
Westville, Durban, South Africa

Tel: +27 73 459 9126
Email: - snf_olatunji@ymail.com

Supplementary Table 1: Retention time and qualifying and quantifying ion characteristics of 3-DDTs and 6-PCB congeners

Compound	Quantifying ion	Retention time (min)	SRM Parent mass	SRM Product mass	Collision energy
4,4'-DDE	Qualify ion (Q1)	18.61	316	246	20
4,4'-DDE	Quantify ion (Q2)	18.61	318	248	20
4,4'-DDD	Qualify ion (Q1)	19.29	235	165	22
4,4'-DDD	Quantify ion (Q2)	19.29	237	199	15
4,4'-DDT_d8	Qualify ion (Q1)	19.41	243	173	25
4,4'-DDT_d8	Quantify ion (Q2)	19.41	245	173	25
4,4'-DDT	Qualify ion (Q1)	19.46	235	165	22
4,4'-DDT	Quantify ion (Q2)	19.46	235	199	16
PCB_110	Qualify ion (Q1)	18.75	324	254	24
PCB_110	Quantify ion (Q2)	18.75	326	256	24
PCB_118	Qualify ion (Q1)	19.30	324	254	26
PCB_118	Quantify ion (Q2)	19.30	326	256	26
PCB_138	Qualify ion (Q1)	19.15	360	290	18
PCB_138	Quantify ion (Q2)	19.15	360	325	18
PCB_149	Qualify ion (Q1)	19.65	358	288	20
PCB_149	Quantify ion (Q2)	19.65	360	290	20
PCB_153	Qualify ion (Q1)	20.09	358	288	22
PCB_153	Quantify ion (Q2)	20.09	360	290	22
PCB_180	Qualify ion (Q1)	21.31	394	324	30
PCB_180	Quantify ion (Q2)	21.31	396	326	30

Supplementary Table 3: Limit of detection (LOD) and limit of quantitation (LOQ) of 6-PCBs and 3-DDTS

Compound				Sensitivity		Linearity
	RT	Standard deviation (σ)	Slope	LOD (µg/L)	LOQ (µg/L)	Coefficient of regression R^2
4,4’-DDE	18.61	1.09	83900.3	0.028	0.084	0.9943
4,4’-DDD	19.29	3.42	111684	0.052	0.156	0.9990
4,4’-DDT	19.46	3.28	111684	0.016	0.048	0.9943
PCB_110	18.75	5.52	24427.4	0.075	0.225	0.9943
PCB_138	19.30	1.37	24427.4	0.018	0.054	0.9943
PCB_118	19.15	2.50	24427.4	0.034	0.102	0.9943
PCB_149	19.65	1.65	24427.4	0.022	0.066	0.9943
PCB_153	20.09	1.64	24427.4	0.022	0.066	0.9943
PCB_180	21.31	1.98	24427.4	0.027	0.081	0.9943

Supplementary Table 3: Average recovery of the 3-DDTs, 6-PCBs and internal standard 4,4'-DDT_d8 from spiked solution

	% recovery at 5 (ng/g) spike	% recovery at 10 (ng/g) spike	% recovery at 25 (ng/g) spike
4,4-DDD	68.20±2.12	75.84±4.16	83.13±5.81
4,4-DDE	83.36±3.91	85.42±2.84	78.90±6.36
4,4-DDT	77.51±1.96	80.90±3.07	89.20±4.18
4,4'-DDT_d8	71.75±4.84	90.09±3.92	81.22±4.17
PCB_110	76.05±4.29	87.29±6.05	79.98±4.19
PCB_118	79.47±3.90	81.92±4.37	76.02±5.42
PCB_138	85.91±6.46	75.09±3.99	82.45±5.22
PCB_149	79.82±5.78	79.42±5.05	78.73±4.85
PCB_153	83.06±4.45	94.29±6.29	86.50±5.29
PCB_180	78.12±6.19	80.26±4.61	91.63±4.08
